# Disease-associated mutations of claudin-19 disrupt retinal neurogenesis and visual function

**DOI:** 10.1038/s42003-019-0355-0

**Published:** 2019-03-25

**Authors:** Shao-Bin Wang, Tao Xu, Shaomin Peng, Deepti Singh, Maryam Ghiassi-Nejad, Ron A. Adelman, Lawrence J. Rizzolo

**Affiliations:** 1 Department of Surgery, Yale University, PO Box 208062, New Haven, CT USA; 2 Department of Ophthalmology, Yale University, 40 Temple Street, New Haven, CT USA; 3 Aier School of Ophthalmology, Central South University, 198 Furong Middle Ave Section 2, Tianxin District, Changsha, China; 4 Present Address: Center for Advanced Vision Science, Department of Ophthalmology, School of Medicine, University of Virginia, Charlottesville, VA 22908 USA; 5 Present Address: Department of Ophthalmology, The Schepens Eye Research Institute, Massachusetts Eye and Ear, Harvard Medical School, 20 Staniford St., Boston, MA 02114 USA

## Abstract

Mutations of claudin-19 cause Familial Hypomagnesaemia and Hypercalciuria, Nephrocalcinosis with Ocular Involvement. To study the ocular disease without the complications of the kidney disease, naturally occurring point mutations of human CLDN19 were recreated in human induced pluripotent cells or overexpressed in the retinae of newborn mice. In human induced pluripotent cells, we show that the mutation affects retinal neurogenesis and maturation of retinal pigment epithelium (RPE). In mice, the mutations diminish the P1 wave of the electroretinogram, activate apoptosis in the outer nuclear layer, and alter the morphology of bipolar cells. If mice are given *9-cis*-retinal to counter the loss of retinal isomerase, the P1 wave is partially restored. The ARPE19 cell line fails to express claudin-19. Exogenous expression of wild type, but not mutant claudin-19, increases the expression of RPE signature genes. Mutated claudin-19 affects multiple stages of RPE and retinal differentiation through its effects on multiple functions of the RPE.

## Abstract (web summary)

Shao-Bin Wang and Tao Xu et al. show that a disease-causing effect of *claudin-19* mutations is due to a defective visual cycle from a decreased expression of *RPE65*. This study explains why *claudin-19* mutations alter the expression of RPE signature genes and impair vision as well as the permeability of tight junctions.

## Introduction

Claudin-19, encoded by the *CLDN19* gene, is a transmembrane protein that determines the permeability and semi-selectivity of tight junctions in the retinal pigment epithelium (RPE), distal tubules and collecting ducts of the kidney, and myelin sheath of myelinated peripheral nerves^1–4^. Mutations in CLDN19 or CLDN16 cause the renal disease familial hypomagnesemia with hypercalciuria and nephrocalcinosis (FHHNC)^5–7^. FHHNC is a family of autosomal recessive disorders that are characterized by excessive urinary loss of magnesium and calcium, nephrocalcinosis, and progressive renal failure. CLDN16 mutations do not affect the eye, which is consistent with its lack of expression in ocular tissues^2^. However, CLDN19 is expressed in human eye, and CLDN19 mutations add ocular involvement to FHHNC (FHHNCOI)^6–8^. Mutations of CLDN19 gives rise to a puzzling array of ocular defects with incomplete penetrance that ranges from no effect to one or more of a variety of defects^7,9–12^. When severe, symptoms appear in infants ~4–9 months old, including bilateral macular coloboma, chorioretinal degeneration, nystagmus, strabismus, and visual loss.

To date, ~16 mutations have been described for CLDN19. This study focuses on mutation of the coding sequence G59A (amino acid sequence, G20D) located in exon 1 and C241T (amino acid sequence, R81W) located in exon 2^9–11^. We chose the G20D and R81W mutations because they are examples of more severe (G20D) and less severe (R81W) disease^11^. Although FHHNCOI is reported as a recessive genetic disorder, some p.G20D affected patients are heterozygous or compound heterozygous when combined with other mutations^9^. Both G20D and R81W patients have typical FHHNCOI symptoms in their childhood^11^. The pathogenesis of the ocular defects in FHHNCOI remains elusive.

It is unclear why mutation of an RPE gene would have such diverse effects on ocular development. In mice, claudin-19 was found in the kidney and Schwan cells of the peripheral nervous system, but was absent from the oligodendrocytes of the central nervous system^4^. To study the effects of a CLDN19 mutation in the absence of renal effects or effects on the peripheral nervous system, we followed three strategies. First, CRISPR/Cas9 (clustered regularly interspaced short palindromic repeats/CRISPR-associated protein 9) was used to create a G20D mutation in a human induced pluripotent stem cell line. Second, the G20D and R81W mutations were overexpressed in the retina of C57BL/6J mouse pups. Third, the two mutant and wild-type genes were exogenously expressed in a human RPE cell line, ARPE-19, that does not express CLDN19 on its own^3^, and in highly differentiated primary cultures of human fetal RPE^2,13–15^.

We demonstrate multiple effects of claudin-19 on the development and function of the RPE and neurosensory retina. Besides its role in barrier function, claudin-19 regulates the expression of RPE signature genes^16,17^ that include visual cycle proteins and retinal neurotrophic factors.

## Results

### CLDN19^G20D^ disrupts retinal neurogenesis in culture

**Fig. 1:**
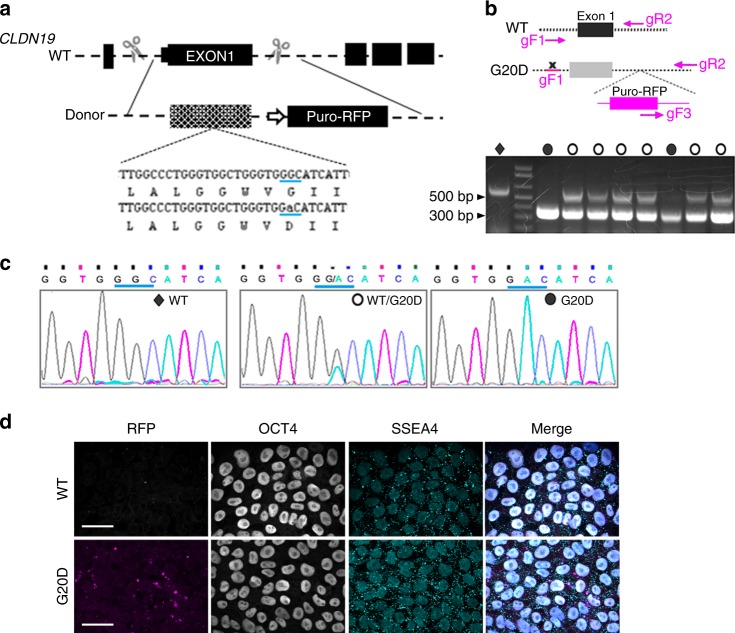
Strategy for creating mutants and expressing them in two-dimensional (2D) cultures. **a** Schema for introducing the G20D mutation into exon 1 using CRISPR/Cas9 (clustered regularly interspaced short palindromic repeats/CRISPR-associated protein 9) technology. The strategy includes adding a puromycin-red fluorescent protein (RFP) gene with a CAG promoter into the following intron. **b** The schema indicates the strategy for PCR-based genotyping of CLDN19 human induced pluripotent cell clones, and the gel shows the result. The uncropped gel is in Supplementary Figure 1. The gF1 primer binds within the promoter region; gR2 primer binds within intron 1; gF3 primer binds within the donor plasmid. For homozygous wild-type (WT) clones, the gF1/gR2 primer combination would yield a product of 550 base pairs. For homozygous clones with an insert, the gF3/gR2 primer combination would dominate to yield a 300-base pair product. Heterozygotes would yield both products. **c** DNA sequencing confirmed the genotype. **d** Confocal images of S34 (WT) and RFP-positive (G20D) clones demonstrated expression of the pluripotency markers SSEA4 (in the cytoplasm) and OCT4 (in the nucleus), as revealed by indirect immunofluorescence imaging. The merge images include 4′,6-diamidino-2-phenylindole (DAPI) to indicate cell nuclei. Diamonds indicate wild type, open circles heterozygote and closed circles homozygote. Scale bar, 20 µm. Supporting data are included in Supplementary Information

To investigate the impact of CLDN19 mutations on retinal development, we used CRISPR/Cas9-mediated mutagenesis to introduce a naturally occurring patient mutation (G59A → amino acid G20D) into the human induced pluripotent cell line, S34 (Fig. 1a, Supplementary Figure 1). S34 was derived from IMR90–4, a human induced pluripotent cell line widely used to study retinal development^18^. S34 expresses SIX6 with green fluorescent protein (GFP) appended to the C terminus. The SIX6 promoter is activated during the initial differentiation of retinal progenitor cells (RPCs), which allowed us to monitor differentiation by fluorescence microscopy. Additionally, S34-G20D expresses a puromycin-RFP reporter. The red fluorescent protein (RFP)-positive clones were selected, as described in the Methods. Briefly, homozygous and heterozygous clones were selected by limiting dilution and PCR (Fig. 1b). Clones were further validated by sequencing genomic DNA (Fig. 1c). All the selected clones show similar expression of pluripotent markers, OCT4 and SSEA4, compared with S34 cells (Fig. 1d).

**Fig. 2:**
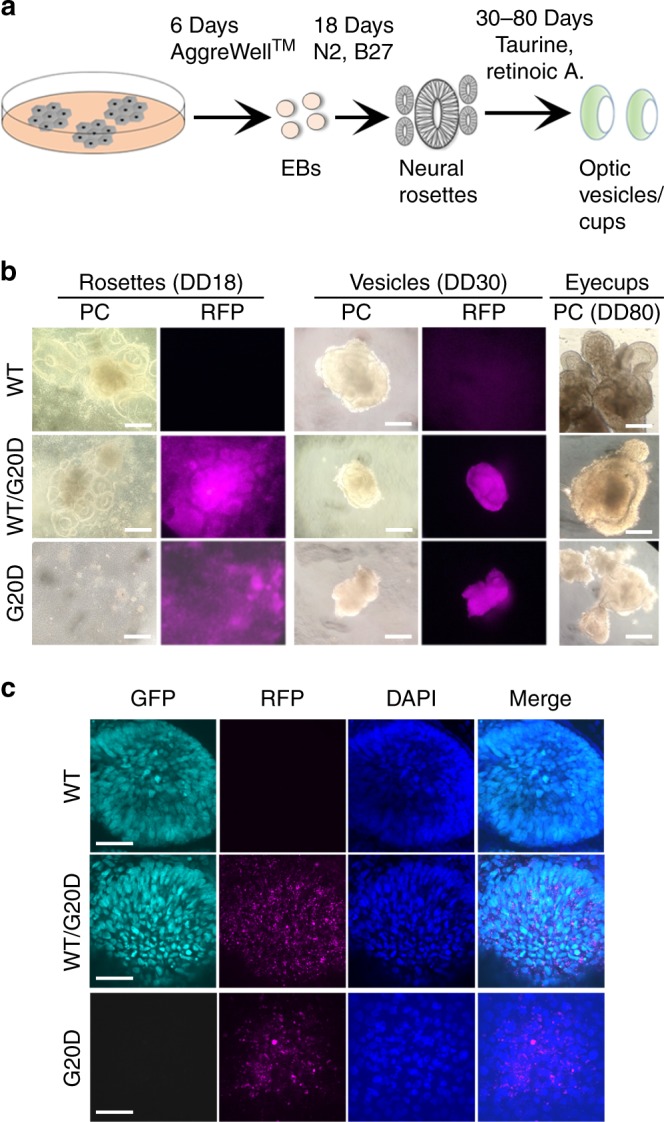
CLDN19^G20D,G20D^ disrupted retinal neurogenesis of human induced pluripotent cells. **a** Schema for differentiating optic vesicles and cups. **b** Live cell imaging: Phase contrast (PC) images show the normal differentiation of neural rosettes and optic vesicles for CLDN19^WT,WT^ and CLDN19^WT,G20D^, and the inability of CLDN19^G20D,G20D^ to form these structures. Fluorescence microscopy confirms the expression of puromycin-red fluorescent protein (RFP) in transduced clones. **c** Confocal images of cultures fixed in paraformaldehyde on 30 days of differentiation (DD30): CLDN19^WT,WT^ and CLDN19^WT,G20D^ expressed SIX6-GFP, an early marker of retinal differentiation, but CLDN19^G20D,G20D^ did not, despite the expression of puromycin-RFP. Scale bar (**b**) 500 µm and (**c**) 20 µm

**Fig. 3:**
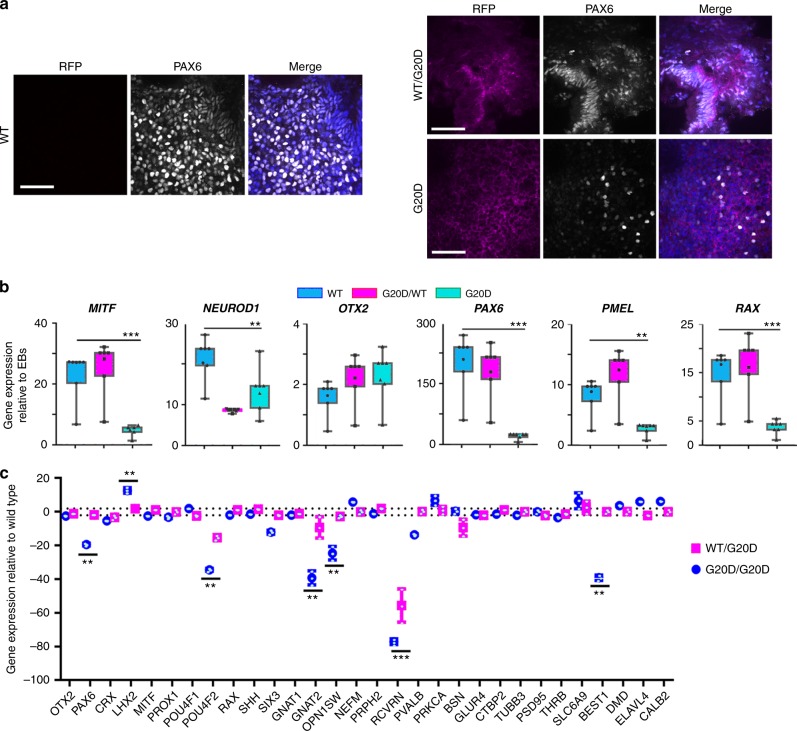
CLDN19^G20D,G20D^ down-regulated the expression of markers for retinal development. Clones were analyzed on 30 days of differentiation (DD30) (**a**, **b**) or DD80 (**c**). **a** Confocal imaging. Optic vesicles expressed PAX6 in CLDN19^WT,WT^ and CLDN19^WT,G20D^ clones but not CLDN19^G20D,G20D^. Puromycin-red fluorescent protein (RFP) was evident in the transduced clones. **b** Expression of messenger RNAs (mRNAs) was determined by quantitative-real-time RT-PCR (qRT^2^-PCR), normalized to glyceraldehyde 3-phosphate dehydrogenase (GAPDH) and compared to expression in undifferentiated embryoid bodies (EBs). **c** Expression of mRNAs was normalized to GAPDH and compared to expression in clones of CLDN19^WT,WT^. Scale bar, 20 µm; error bars: SEM for three isolates each of two (G20D) or three (G20D/WT) clones. **P < 0.01, ***P < 0.001

Following the protocol to differentiate optic vesicles, all three CLDN19^WT/G20D^ clones expressed RFP and GFP, but the two CLDN19^G20D/G20D^ clones expressed only RFP (Fig. 2). Only CLDN19^WT/G20D^ clones formed neural rosettes, optic vesicles, and optic cups. CLDN19^G20D/G20D^ clones formed solid aggregates that failed to form retina-like lamina. All clones expressed CLDN19 messenger RNA (mRNA) in amounts within 4× of CLDN19^WT/WT^. Only CLDN19^WT/WT^ and CLDN19^WT/G20D^ up-regulated the expression of differentiation markers for optic vesicles, such as microphthalmia-associated transcription factor (MITF), paired box 6 (PAX6), premelanosome protein (PMEL), and RAX (Fig. 3). After 80 days of differentiation (DD80), a quantitative-real-time RT-PCR (qRT^2^-PCR) array was used to compare the effects of CLDN19^G20D^ on the expression of retinal genes relative to CLDN19^WT/WT^. The array included 29 genes that are expressed by various retinal cell types. CLDN19^WT/G20D^ expressed all these mRNAs at levels comparable to wild type except recoverin, which was down-regulated 60×. Besides recoverin, CLDN19^G20D/G20D^ also down-regulated PAX6, POU4F2, GNAT2, and OPN1SW. The down-regulation of recoverin was confirmed by indirect immunofluorescence (Supplementary Figure 2).

### CLDN19^G20D^ disrupts the differentiation of RPE in culture

**Fig. 4:**
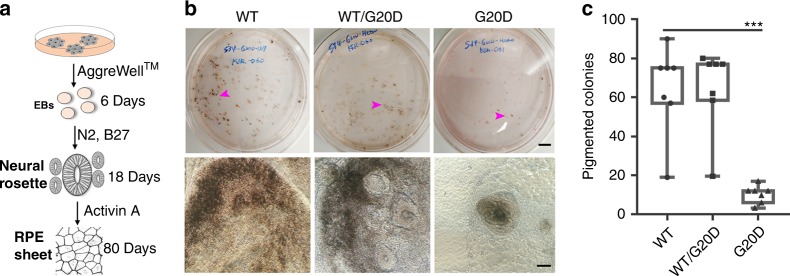
The CLDN19^G20D/G20D^ clones form pigmented clusters inefficiently. **a** The schematic drawing illustrates how retinal pigment epithelium (RPE) was differentiated from neural rosettes. **b** Low-power and high-power images of pigmented colonies present on 80 days of differentiation (DD80). Scale bars: Top row, 1.0 cm; bottom row, 500 µm. Arrowheads indicate pigmented colony. **c** On DD80, pigmented colonies generated by RPE differentiation were quantified. The number of pigmented colonies formed by CLDN19^WT/G20D^ was similar to wild type (ns, not statistically significant). CLDN19^G20D/G20D^ produced significantly fewer. Mean values represent number of pigmented colonies in each dish (4 dishes). Error bars ± SEM; ***P < 0.001

**Fig. 5:**
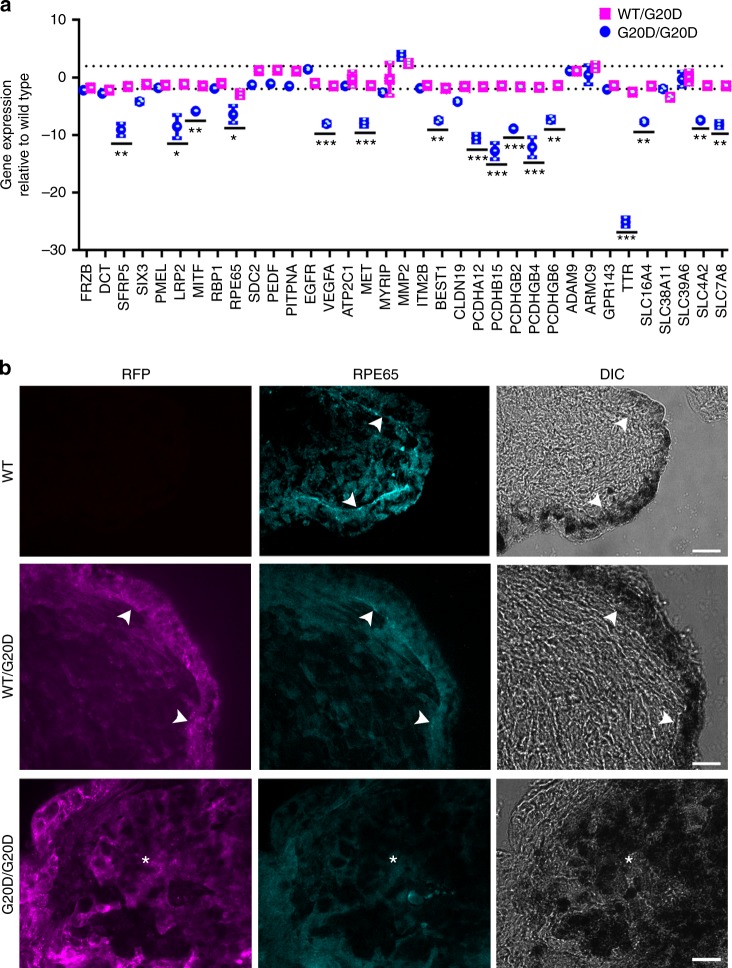
Like wild type, pigmented colonies formed by CLDN19^WT/G20D^ exhibited normal morphology and gene expression, but CLDN19^G20D /G20D^ colonies were malformed and under-expressed retinal pigment epithelium (RPE) signature and maturation genes. Analyses were performed on 80 days of differentiation (DD80). **a** A quantitative-real-time RT-PCR (qRT^2^-PCR) analysis compared expression relative to wild type. Error bars: SEM for three clones (**P < 0.01, ***P < 0.001). Dotted line, ±2× difference. **b** Immunostaining of RPE65 (cyan). Red fluorescent protein (RFP), expression of puromycin-RFP (magenta). Arrowheads indicate interface of RPE with the neurosensory retina; asterisks indicate cluster of pigmented cells. Scale bar, 20 µm

The preceding data implicated genes that are found in RPE and retinal precursors, such as PAX6, PMEL, and MITF that are important for RPE differentiation^19^. To pursue the hypothesis that the differentiation of RPE is impaired, Activin A was used to foster the differentiation of human induced pluripotent cells into RPE (Fig. 4)^20^. Pigmented colonies appeared 60 to 80 days post induction. For CLDN19^WT/WT^, 75 of 800 (~9.3%) colonies were pigmented after 60 days. For CLDN19^WT/G20D^, 73 of 800 (~9.1%) colonies were pigmented. For CLDN19^WT/G20D^, only 12 of 800 (1.45%) colonies were pigmented. A qRT^2^-PCR array of RPE signature and maturation genes revealed that CLDN19 mutant pigmented cells express lower amounts of the mRNA for MITF, solute carriers (SLC7A8, SLC4A2, SLC16A4), TTR, protocadherins (PCDHGB, PCDHA), BEST1, and RPE65 (Fig. 5). RPE65 (retinal isomerase) and TTR (transthyretin) are critical components of the visual cycle in RPE cells^21^. We examined the morphology and protein expression of the RPE monolayers. Phase contrast images revealed a monolayer of RPE lining the presumptive neurosensory retina only in cultures derived from CLDN19^WT/WT^ and CLDN19^WT/G20D^ (Fig. 5b). A disorganized cluster of pigmented cells was observed in CLDN19^G20D/G20D^. RPE65 was detected in the pigmented cells of only CLDN19^WT/WT^ and CLDN19^WT/G20D^ derivatives. The pigmented melanin granules were concentrated in the basal pole of the cells, with the RPE65 observed in the apical pole. The apparent polarity of the RPE65 might be due to quenching of the fluorescent signal by the melanin granules.

### CLDN19 mutations induce retinal degeneration and visual loss

**Fig. 6:**
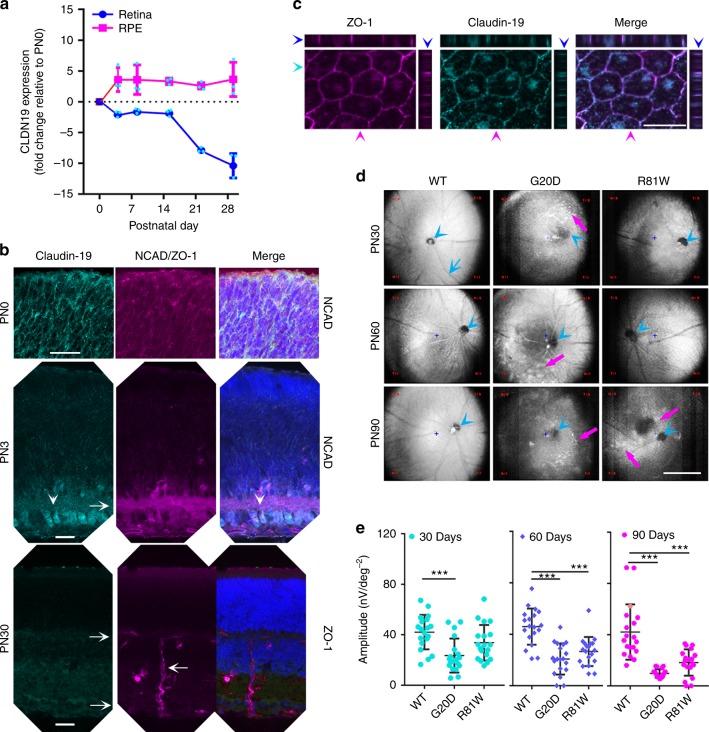
In mice, CLDN19 is expressed in the retinal pigment epithelium (RPE), newborn retina, and overexpression of human CLDN19 mutants reduced the light response. **a** Quantitative-real-time RT-PCR (qRT^2^-PCR) detected endogenous CLDN19 messenger RNA (mRNA) in RPE and in the developing retina from postnatal days 0 to 21 (PN0 to PN21). Total RNA was isolated from the retinae, or RPE sheets, and pooled from 3 mice; *n* = 3 experiments. **b** Immunostaining of the neurosensory retina revealed endogenous claudin-19 initially present throughout the retina and later enriched in retinal ganglion cells (white arrowheads) on PN3. Claudin-19 was undetected on PN30 in the neurosensory retina. The inner plexiform layer was revealed by N-cadherin (NCAD; magenta) on PN3, and zonula occludens-1 (ZO-1; magenta) revealed the inner and outer retinal vascular beds (rightward arrows) on PN30. A vessel connects the two beds (leftward arrow). Scale bar, 20 µm. **c** RPE monolayer on P30 demonstrates that endogenous claudin-19 and ZO-1 co-localize in the tight junction. The *XZ* plane is at the top of the *XY* plane and the *YZ* plane is at the right. Blue arrowheads indicate location of the *XY* plane; cyan arrowheads indicate location of the *XZ* plane; magenta arrowheads indicate location of the *YZ* plane. Scale bar, 20 µm. **d** Adeno-associated virus (AAV) viral vectors that express exogenous CLDN19^WT^, CLDN19^R81W^, or CLDN19^G20D^ were injected into the subretinal space at PN0. Fundus (posterior retina) imaging was acquired on PN30, PN60, and PN90. Magenta arrows indicate hypo- or hyper- dense areas caused by the expression of mutated CLDN19; blue arrowheads show optic nerve head; blue arrow indicates a branch (or tributary) of the central ophthalmic artery (or vein). Scale bar, 500 µm. **e** Quantification of multifocal-electroretinogram P1 amplitudes at PN30, PN60, and PN90. Data were acquired from 9 different loci in 3 mice at each time point. Error bars ± SEM; ***P < 0.001

Before we could use a mouse model to examine how claudin-19 affects retinal differentiation in vivo, we had to resolve the confusion about which claudins are expressed in mice^4,22^. To confirm claudin-19 is expressed, cell extracts were prepared from the RPE and developing neurosensory retina of mouse pups. Messenger RNA for claudin-19 was evident in both tissues at birth (postnatal day 0 (PN0)) and continued to be expressed in RPE through adulthood (Fig. 6a–c). Whereas expression in the RPE increased ~4× after PN3, expression in the neurosensory retina decreased after PN14. At PN0, indirect immunofluorescence staining revealed that claudin-19 was expressed in RPE and throughout the retinal precursors. By PN3, when the ganglion cell layer appeared, claudin-19 was restricted to that layer. By PN30, claudin-19 was undetected in the neurosensory retina. The retinal vasculature, as visualized by antibodies to zonula occludens-1 (ZO-1), helped delineate the layers of the adult retina. In RPE, claudin-19 was found in a circumferential band at the apical end of the lateral plasma membrane. There, it co-localized with ZO-1, a structural protein of tight junctions.

To investigate the ocular effects of naturally occurring CLDN19 mutations without the complications of kidney or peripheral nerve disease^4^, adeno-associated virus (AAV) vectors were injected into the subretinal space of PN0 mice to transduce the RPE and retinal progenitor cells (Fig. 6d, e). The AAV vectors encoded one of the following human CLDN19 genes under the control of a CAG promoter: CLDN19^WT^, CLDN19^G20D^, or CLDN19^R81W^. The mice were subsequently examined by fundoscopy and multifocal electroretinography (mfERG) on PN30, PN60, and PN90. The fundoscopic images of eyes transduced with CLDN19^WT^ appeared normal. Eyes transduced with CLDN19^G20D^ exhibited hyper-intense spots by PN30 and hypo-intense regions by PN90. Eyes transduced with CLDN19^R81W^ exhibited the same, although milder, pathology with a slower time-course. The fundus morphology appeared normal on PN30 and PN60 but exhibited hyper- and hypo-intense regions on PN90. Corresponding mfERG recordings were obtained in light-adapted animals and the amplitude of the P1 wave was determined. The P1 wave reflects transfer of the nerve impulse from photoreceptors to bipolar cells and other interneurons. For wild type, P1 = 42 ± 13 nV deg^−2^ and remained stable for the duration of the experiment. By PN30, light response for CLDN19^G20D^ mice was reduced (P1=23 ± 13 nV deg^−2^), but the response for CLDN19^R81W^ mice (P1=33 ± 15 nV deg^−2^) was not significantly different from wild type (*p* > 0.05). For CLDN19^G20D^ and CLDN19^R81W^ the light response progressively decreased with time. Though overexpression of the human wild-type gene had no discernable effect, overexpression of the human mutants overwhelmed the effects of the endogenous mouse gene.

**Fig. 7:**
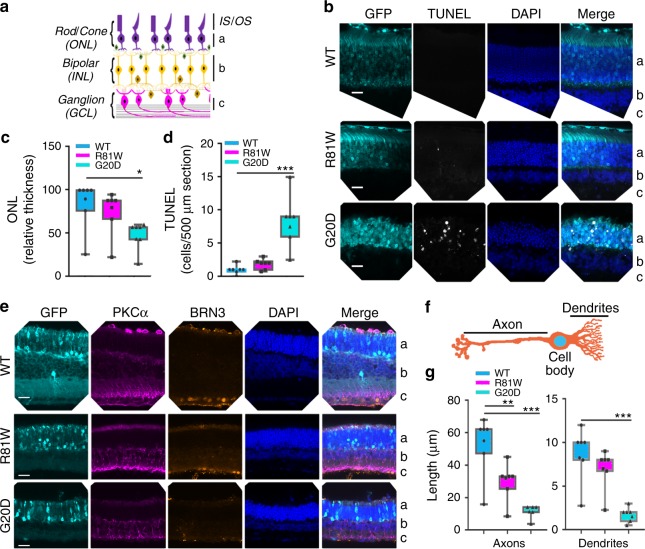
CLDN mutants induced degeneration of the outer nuclear layer (ONL) and the remodeling of bipolar cells in the inner nuclear layer (INL). The ganglion cell layer (GCL) layer was unaffected. Data were collected 30 days after transduction on postnatal day 0 (PN0). Green fluorescent protein (GFP) revealed transduced cells only in the ONL (**b**–**e**). The retinal pigment epithelium (RPE) is not included in the images. **a** Schema of the retina defines the "**a**, **b**, **c**" labels used in (**b**, **e**). **b** TUNEL (terminal deoxynucleotidyl transferase dUTP nick end labeling) staining indicated that photoreceptor cell death induced by CLDN19^G20D^>CLDN19^R81W^>CLDN19^WT^ and was only observed in the ONL. **c**, **d** The impression in **b** was confirmed by quantification of apoptosis and ONL thickness. **e** Immunofluorescence staining revealed ganglion cell nuclei (BRN3) and the neurites of bipolar cells (protein kinase Cα (PKCα)). **f** Schematic diagram of a bipolar cell. The bars indicate where measurements were made for axon and dendritic length. **g** Quantification of axon and dendritic length in the retinal bipolar cell layer. The length of axons and dendrites was reduced in retinas transduced with mutated CLDN19. Measurements were made from four different loci from three mice in each group. IS photoreceptor inner segment, OS photoreceptor outer segment, ONL outer nuclear layer, OPL outer plexiform layer, INL inner nuclear layer, IPL inner plexiform layer, RGC retinal ganglion cell layer. Error bars, SEM (*P < 0.05, **P < 0.01,***P < 0.001). Scale bar, 20 µm

The retina degenerated in parallel with the effects on mfERG. On PN30, the CLDN19^WT^ and CLDN19^R81W^ transduced mice exhibited relatively normal gross retinal structure (Fig. 7b–e). CLDN19^G20D^ transduced mice had a significantly thinner outer nuclear layer (*p* < 0.05). Rhodopsin properly localized to the inner and outer segments in the mutated photoreceptors (Supplementary Figure 3). The TUNEL (terminal deoxynucleotidyl transferase dUTP nick end labeling) assay revealed significantly higher apoptosis (*p* < 0.001) in the outer nuclear layer of the CLDN19^G20D^ mice (Fig. 7b–d). No apoptosis was observed in the inner nuclear layer or ganglion cell layers.

Retinal degeneration often includes the remodeling of retinal bipolar cells^23,24^. Remodeling of bipolar cells due to CLDN19 mutations was evident from the altered length of the axon and dendrites (Fig. 7e–g). At PN30, CLDN19^WT^ mice exhibited normal morphology of retinal bipolar cells. The length of bipolar axons from the inner nuclear layer to inner plexiform layer was ~60 µm and the dendrites from photoreceptor layers to soma of bipolar cells was ~10 µm. In contrast, both CLDN19^G20D^ and CLDN19^R81W^ mice had shortened retinal bipolar cells with the axon length: ~40 µm for CLDN19^R81W^ and ~15 µm for CLDN19^G20D^. The length of bipolar dendrites was ~8 µm for CLDN19^R81W^ and ~2 µm for CLDN19^G20D^ (Fig. 7e–g).

**Fig. 8:**
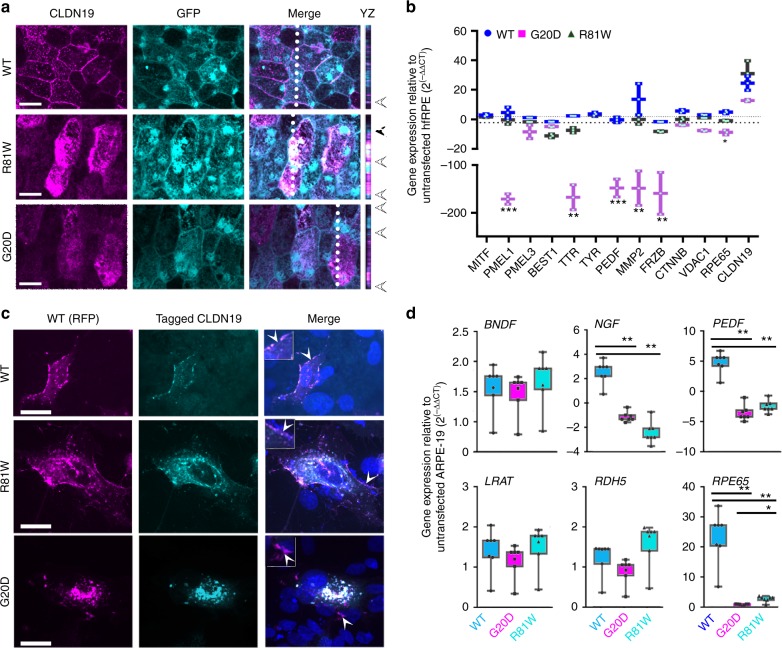
CLDN19 mutations interfered with the intracellular transport of endogenous claudin-19 and down-regulated some retinal pigment epithelium (RPE) signature genes. **a** A mixture of AAV-GFP and AAV-CLDN19 viral vectors were injected into the subretinal space on postnatal day 0 (PN0) to co-express green fluorescent protein (GFP) and either CLDN19^WT^, CLDN19^G20D^, or CLDN19^R81W^. The RPE monolayer was analyzed on PN30. The *XZ* plane displays the apical half of the cell imaged along the dotted line. Arrowheads at the right indicate the position of apical junctions. GFP indicated that most cells of the monolayer were transduced. Wild-type (WT) claudin-19 localized primarily to the tight junction, but G20D was mostly found in intracellular compartments. R81W displayed an intermediate phenotype. **b** Effect of claudin-19 on gene expression in human fetal RPE. Plasmid versions of the viral vectors were used to transfect human fetal RPE. Expression of WT claudin-19 had minimal effects, but G20D and to a lesser extent R81W down-regulated the expression of a subset of RPE signature genes; *n* = 3 cultures. **c** Effect of mutant CLDN19 on the intracellular transport of claudin-19. A red fluorescent protein (RFP) coding sequence was added to the 5’ end of claudin-19. In a second set of plasmids, a FLAG-tag (wild type) or HA-tag (mutants) was added to the 5’ end. The RFP and one of the antigen-tagged plasmids were co-transfected into ARPE-19 cells. Cells that were co-transfected are shown. Most of the wild-type claudin-19 reached the plasma membrane and the RFP and FLAG-tagged versions co-localized in plasma membrane and internal compartments. In the R81W cells, R81W and wild type also co-localized in the plasma membrane with an increase in the relative amount found in internal compartments. In the G20D cells, mutated claudin-19 did not reach the cell surface and most wild-type claudin-19 co-localized with the mutated claudin-19 in internal compartments. **d** Effect of claudin-19 on gene expression in ARPE-19. Expression of messenger RNAs (mRNAs) in transfected ARPE-19 was determined by quantitative-real-time RT-PCR (qRT^2^-PCR), normalized to glyceraldehyde 3-phosphate dehydrogenase (GAPDH), and compared to expression in untransfected *n* = 4 culture ARPE-19. Dashed lines indicate a 2× difference. Arrowheads indicate regions enlarged 4× in the insets of the merged image. Scale bar, 20 µm. Error bars ± SEM. *P < 0.05, **P < 0.01, ***P < 0.001

There was no overt effect on the RPE of mice in vivo. The cells maintained their characteristic polygonal morphology, and there was no evidence of apoptosis, either by the TUNEL assay or increased expression of Caspase 3 (Supplementary Figure 4). Wild-type human claudin-19 localized normally to the lateral membranes of RPE cells and a few internal vesicles. However, both CLDN19^G20D^ and CLDN19^R81W^ mis-localized to internal compartments (Fig. 8a). To study the effect of the mutations on gene expression, we turned to human fetal RPE, but we were unable to transduce differentiated cultures with AAV vectors. Instead, we transfected suspensions of human fetal RPE with the corresponding plasmids, plated the cells, and analyzed the cultures 1 week after they became confluent (Fig. 8b). At this time, the tight junctions and polarity of the cells had insufficient time to mature, but effects on gene expression were observed. Using primers that would amplify all CLDN19 variants, the expression of CLDN19 in the cultures increased 15–30× over endogenous CLDN19 alone. CLDN19^G20D^ down-regulated a subset of the signature genes. These genes included: FRZB, MMP2, PEDF, PMEL1, RPE65, and TTR.

To study the effects of claudin-19 in greater detail, we used an RPE cell line that fails to express endogenous claudin-19, ARPE-19. When CLDN19^WT^ was expressed in ARPE-19, expression of mRNAs increased for RPE signature genes^3,25^. In the present study, we confirmed that expression of CLDN19^WT^ up-regulated BEST1, NGF, PEDF, RPE65, SIX3, SFRP5, and TTR. ARPE-19 was co-transfected with two plasmids that encoded claudin-19 with a fluorescent or antibody tag added to the N terminus. In the first plasmid, RFP was added to CLDN19^WT^ to form RFP-CLDN19^WT^. The second plasmid encoded one of the following: Flag-CLDN19^WT^, HA-CLDN19^G20D^, or HA-CLDN19^R81W^ (Fig. 8c, d). As expected, the two versions of claudin-19^WT^ co-localized in the lateral membranes with some claudin-19 observed in internal vesicles, as reported for untagged claudin-19^3^. When RFP-CLDN19^WT^ was co-expressed with HA-CLDN19^G20D^, the two claudins co-localized in an internal vesicular compartment. A weak signal for RFP-claudin-19^WT^ alone was observed in tight junctions. When co-expressed with CLDN19^R81W^, a milder phenotype was observed. A portion of claudin-19^R81W^ co-localized with RFP-claudin-19^WT^ in the lateral membranes; the remainder was found in internal vesicles. The fluorescent signal of the lateral membrane was whitish magenta, which indicates a strong magenta signal from WT-RFP relative to the R81W, cyan signal. The internal structures were white, which indicates a higher R81W/WT ratio in the internal pool. The mutant claudins were unable to upregulate the expression of mRNAs that were up-regulated by CLDN19^WT^, including nerve growth factor (NGF), pigment epithelium-derived factor (PEDF), and RPE65.

### CLDN19 mutations adversely affect the visual cycle in RPE

**Fig. 9:**
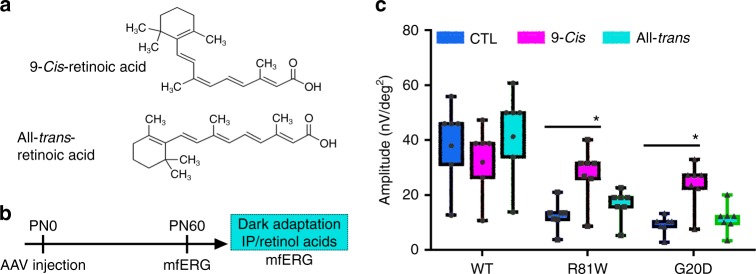
Administration of 9-*cis-*retinal partially restored visual function. **a** The structure of 9-*cis-*retinoic and all-*trans-*retinoic acid. **b** Schema of the protocol for expressing exogenous CLDN19, obtaining baseline multifocal electroretinograms on P60, and administering the light-sensitive retinoids into dark-adapted animals by intraperitoneal injection. **c** Multifocal electroretinograms were obtained in the light to estimate the amplitude of the P1 wave. Error bars, SEM for 4 mice in each group. *P < 0.05

Besides the neurotrophic factors that are regulated directly or indirectly by claudin-19, RPE65 is part of the visual cycle that re-isomerizes all-*trans*-retinal to regenerate 11-*cis*-retinal. To test the hypothesis that an impaired visual cycle contributes to the pathology of FHHNCOI, we attempted to rescue visual function by delivering exogenous retinoids at PN60 via intraperitoneal injection. Multifocal electroretinography recordings were used to measure the P1 wave, which reflects transmission of the light signal from photoreceptors to inner neurons. Prior to treatment, this response to light was diminished in CLDN19^R81W^ transduced mice and absent in CLDN19^G20D^ transduced mice (Fig. 9). After intraperitoneal injection of 9-*cis*-retinal, a functional analog of the visual chromophore 11-*cis*-retinal, the amplitude of the P1 wave increased in CLDN19^G20D^ and CLDN^R81W^ mice. In control experiments, all-*trans*-retinal, the precursor of 11-*cis*-retinal, failed to restore a light response.

## Discussion

We developed four models of a human genetic disease that affects the choroid, RPE, and neurosensory retina: human induced pluripotent cells, mice, and two culture models of RPE. The human induced pluripotent cells provide a way to study how the pathology develops during the differentiation of the human retina, although in vitro. The mouse model is suited to study how pathology develops in vivo, and how the mutations affect visual function. Although a CLDN19-knockout mouse has been developed, ocular effects were not described^4^. We did not pursue this model, because the analysis would be complicated by the severe kidney and peripheral nerve disease present in these mice. The primary cultures of human fetal RPE have been extensively authenticated for native RPE gene expression and physiologic function^2,13–15^. The ARPE-19 cell line is instructive, because it lacks claudin-19, and portions of the RPE phenotype can be restored by exogenous expression of claudin-19^3^. We combined the strengths of each model by recreating point mutations that have been identified in human patients. We are aided by conservation of claudins, ocular development, and visual function across vertebrate species. Besides the novel signal transduction role described previously^3^, two unanticipated roles for claudin-19 emerged from this study.

Like other claudins, claudin-19 helps determine the tissue-specific barrier properties of the epithelial tight junctions in which it is found. Claudin-19 forms a “tight” rather than “porous” junction that is slightly cation selective^2,26^. Exogenous expression of CLDN19 decreased permeability and increased cation selectivity of pig kidney cell lines, whereas expression of CLDN19^G20D^ had no effect^27^. Several claudins share the properties of low permeability and cation selectivity. RPE expresses a handful of those claudins, but in amounts too low to create a barrier without claudin-19^2^. In principal, these could be up-regulated to compensate for dysfunctional claudin-19. However, one reason for the redundant barrier properties of the 24, or more, claudins may lie in the signal pathways that a given claudin activates^3,28–31^. In this hypothesis, increasing the expression of other RPE claudins might restore barrier function, but without inducing RPE-specific gene expression, as claudin-19 does.

In vitro, mutation of claudin-19 allowed early neurogenesis to occur, but retinal-specific neurogenesis was impaired. The RPE differentiates before other retinal tissues. Impairing the differentiation of RPE might prevent other retinal tissues from differentiating. Alternatively, CLDN19 might play a role in facilitating the transition from early eye field-to-optic vesicle-to-optic cup^32,33^. Our lab has previously demonstrated that the RPE-derived cell line ARPE-19 lacks claudin-19 and has insufficient expression of other claudins to compensate for its absence. ARPE-19 only forms rudimentary tight junctions in our hands^3,34^. Exogenous expression of claudin-19 restored many epithelial properties and affected RPE-related gene expression, in part via an AKT-mediated pathway^3^. We confirmed those studies and demonstrated that the claudin-19 mutants examined here failed to increase the transepithelial electrical resistance above the 15–20 Ω cm^2^ that typifies a monolayer of fibroblasts or upregulate the expected genes. Instead they down-regulated PEDF and NGF. The latter secretory products are essential for retinal differentiation^35^. The developmental defect in culture was more severe than in patients. In patients, optic vesicles and optic cups must form, because the eye appears normal except for gross maldevelopment of the retinal macula^6–8^. The macula is the spot where light is focused and is responsible for visual acuity in humans. There are little data on subtle changes that claudin-19 mutations might induce distal to the macula.

Mice lack a macula, and the location of observable, focal lesions was variable. In normal mice, the distribution of claudin-19 is like that of the transcription factor, MITF. It is initially expressed in the nascent RPE and RPCs, but gradually becoming restricted to the RPE. The difference is that claudin-19 disappears from RPCs but remains in ganglion cells for 2 weeks. It remains to be determined whether claudin-19 affects gene expression in nascent ganglion cells.

The mouse model avoided the early steps of retinal development. At birth, the eyecup is formed and the maturation of RPE is on-going^36,37^. This time corresponds to DD30 in the human induced pluripotent cell model. Viral vectors can be injected on PN0 into the subretinal space, between the RPE and RPCs. Cells in both layers were infected, but the data indicate that the viral vector only infected the RPCs destined to become photoreceptors, and not cells destined to become inner neurons. Conceivably, retinal ganglion cells would have been transduced if the subretinal space were injected earlier during embryogenesis. Because claudin-19 affects gene expression, prolonged expression of wild-type claudin-19 beyond the time it is normally expressed in the RPCs might adversely affect retinal development. Wild-type claudin-19 had no apparent effect on photoreceptor apoptosis, morphology, or function (as measured by ERG). We cannot rule out that early effects of the mutants on RPCs lead to the shortened morphology of bipolar cells, except to say that claudin-19 was not observed in bipolar cells. Accordingly, we favor the hypothesis that the effects of mutated claudin-19 were likely due to direct effects on the RPE with secondary effects on the several layers of the neurosensory retina. These effects included focal lesions observed by fundus microscopy, apoptosis in the outer nuclear layer, and shortened axons and dendrites of bipolar cells. The hypothesis is supported by the down-regulation of PEDF and NGF, two retinotrophic factors normally secreted by RPE^35^.

Among the affected RPE genes, RPE65 was down-regulated. In the visual cycle, 11-*cis*-retinal, a co-factor for photoreceptor opsins, is converted to all-*trans*-retinal when light is captured by the opsin. The all-*trans-*retinal is recycled by sending it back to the RPE for re-isomerization by RPE65^38–40^. Mutations of RPE65 cause the retinal degeneration, Leber congenital amaurosis, and retinitis pigmentosa^41,42^. A mouse model of this disease is characterized by rapid loss of a light response followed by a slow retinal degeneration^43^. To test the hypothesis that impairment of the visual cycle caused the loss of the electroretinogram response, the visual cycle was circumvented with 9-*cis*-retinal (Fig. 9). The mice were transduced on P0 with mutant CLDN19 and given 9-*cis-*retinal on PN30 after retinal differentiation was complete, but retinal degeneration was partial. The advantage of 9-*cis-*retinal over 11-*cis-*retinal is that it bypasses the RPE portion of the visual cycle^44^. The P1 wave of the electroretinogram indicates that photoreceptors transmitted a signal to inner neurons. The P1 wave was restored by 9-*cis*-retinal, but not all-*trans*-retinal. Because patients with FHNNCOI present with kidney disease earlier than ocular disease, supplementation with *cis*-retinal and neurotrophins might provide a therapy to preserve visual function until gene therapy can be implemented.

**Fig. 10:**
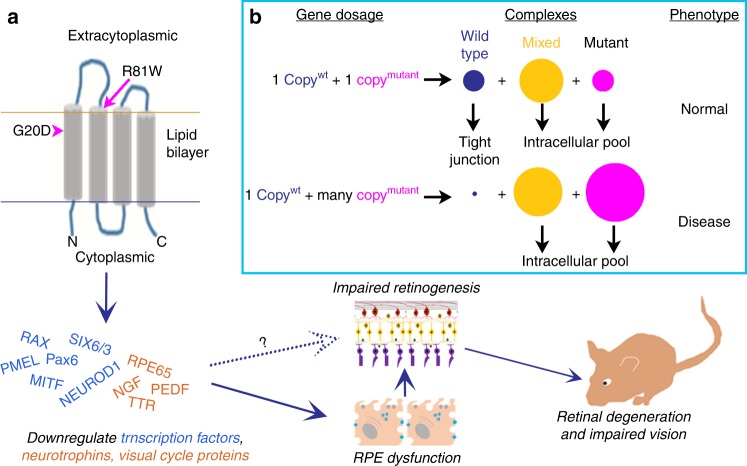
CLDN19 mutations have multiple effects on retinal development. **a** In the human stem cell model, neurospheres formed, but not optic vesicles or eye cups. Expression was repressed for early eye field transcription factors and many retinal pigment epithelium (RPE)-related genes. Once RPE was established in culture or in mice, the overexpression of claudin mutants led to decreased expression of neurotrophins and RPE65, which led to retinal degeneration and impaired visual function. The loss of RPE65 could be compensated by providing animals with 9-*cis-*retinal. The solid arrows indicate how correcting the visual cycle can spare vision, but the effects of decreased secretion of neurotrophins by RPE remain to be studied. The dotted arrow indicates that direct effects of claudin-19 on early retinal development are suggested by the study but remain to be explored. **b** The box suggests a hypothesis whereby a recessive mutation in vivo might lead to a dominant effect when overexpressed. Claudins are known to form complexes, and these are required by claudin-19 to reach the cell surface. Mutant claudin-19 accumulates within the cell and blocks the transport of wild-type claudin-19 from reaching the surface. In heterozygotes, enough wild-type claudin-19 reaches the cell surface to perform its cellular functions (top line). If the mutant is over-expressed, most of the wild-type protein is trapped in internal membranes and the recessive phenotype is observed

The intracellular trafficking of the mutated claudin-19 in RPE was similar to that in the LLC-PK1 kidney cells^27^. In each cell type, G20D was retained in internal compartments with a complete loss of function. Nearby mutations (Fig. 10), R81W (this study) and L90P (LLC-PK1), could reach the tight junction and retain partial function. A matter of confusion is how claudin-19 reaches the cell surface in the culture studies. In the kidney, claudin-19 must be complexed with claudin-16 to reach the cell surface^27,45^. However, human RPE delivers claudin-19 to tight junctions without claudin-16^25^. ARPE-19 and the kidney cell lines, MDCK and LLC-PK1, do not express claudin-16 or claudin-19. Nonetheless, when claudin-19 is exogenously expressed it reaches the tight junctions^2,3,27,45^. It is unknown what proteins might deliver claudin-19 to tight junctions in claudin-16-null cells.

A concern related to intracellular trafficking is that FHHNCOI is a recessive disease. Patients who are heterozygous for G20D manifest disease only if the opposite allele contains a distinct CLDN19 mutation^9^. In our experiments, heterozygous human induced pluripotent cells differentiated normally, but homozygous human induced pluripotent cells did not. The mice expressed two wild-type CLDN19 alleles but exhibited retinal defects in the presence of exogenous CLDN19 mutations. The model in Fig. 10b describes one hypothesis for why the mutations had a dominant rather than a recessive phenotype in this experiment. If claudin-19 needs to be incorporated into a protein complex for intracellular transport, the inclusion of mutant claudin-19 would inhibit the transport of all claudin found in the same complex. In heterozygous patients, such complexes are proposed to contain all wildtype, all mutant, or a mixture of wild type and mutant claudin-19. Because heterozygotes would express equal expression amounts of both alleles, enough complexes of pure wild-type claudin-19 would reach tight junctions to support normal function. Because our mutant constructs are under the control of the powerful CAG promoter^46^, high levels of mutated claudin-19 would overwhelm the wild-type protein.

This study demonstrates that claudin-19 affects retinal development by regulating the expression of a subset of RPE signature genes, including RPE65, PEDF, and NGF. In vitro, this regulatory function occurs in confluent cultures before tight junctions have matured to function as a barrier. The effects of altering the secretion of neurotrophic factors and potential effects on barrier function remain to be explored. The transient expression of claudin-19 in retinal progenitors and nascent retinal ganglion cells is a second avenue of research engendered by this work. The data suggest that exogenous retinoids and neurotrophic factors may be therapeutic candidates to prevent the ocular effects of FHHNCOI disease.

## Methods

### Animals and cell lines

ARPE-19 cells were purchased from the American Type Culture Collection (Manassas, VA). Cultures were used from passages 27 to 32 and within 3 passages of verification by short tandem repeat analysis performed by American Type Culture Collection (Manassas, VA). Culture media and supplements were purchased from Gibco/ThermoFisher Scientific (Waltham, MA) unless otherwise indicated. At each passage, cells were seeded at a density of 1.5 × 10^5^ cells cm^−2^, and cultured with Dulbecco's modified Eagle's medium (DMEM) with 10% heat-inactivated fetal bovine serum (FBS; Atlanta Biologicals, Norcross, GA), 2.0 mM glutamax, penicillin–streptomycin, and 1.0 mM sodium pyruvate. Cultures became confluent after 2–4 days, and the FBS was reduced to 1% to maintain the cultures^3^. HEK293 cells were maintained in DMEM (high glucose) containing 10% FBS and 1:100 (v/v) glutamine penicillin–streptomycin. Human fetal RPE were the generous gift of Arvydas Mamanishkis and Sheldon Miller (National Eye Institute) and cultures were maintained in a serum free medium that promotes their differentiation, as we have described^13^. S34 cells were the generous gift of Karl Wahlin and Donald Zack (Johns Hopkins University) and were derived from the human induced pluripotent stem cell line, IMR90–4 (WiCell, Madison, WI). Cultures were checked monthly for the presence of mycoplasma using the Mycosensor QPCR Kit (Agilent, Santa Clara, CA).

C57BL/6J WT mice were obtained from Jackson Laboratory (Bar Harbor, ME). Both sexes were studied and the data from both were combined. Animal experiments were approved by the institutional animal care and use committee at Yale University (protocol no. 2014–10577).

### Generation of pG20D iPSC line by CRISPR/Cas9-mediated mutagenesis

The guide RNA (gRNA) plasmid targeted the first exon of CLDN19. The oligonucleotides (forward: CACCGTACTGCCCTGTGCCCATTGA; reverse: AAACTCAATGGGCACAGGGCAGTAC) were synthesized by the Keck DNA Facility (Yale University, New Haven, CT). The annealing DNA fragments were inserted into pSpCas9(BB)-2A-GFP (PX458, Addgene, Cambridge, MA)^47^. For construction of the donor plasmid, the homologous arms were amplified using a template of human genome DNA derived from ARPE-19 cells. Upstream arm primers were: forward: CAGTGGGGCCCACTTGAACGTGAGG, reverse: GCCACCCAGGGCCAAGAAGTAGCCC; downstream arm primers were: forward: TGGGTGGACATCATTGCTAGCACAGCC, reverse: ACTGAGTCGGGCTGGCGGGGATGGG. The coding sequence 59G−>A mutant was generated through PCR-based mutagenesis. For the selection of clones, the coding sequence of puromycin-RFP was inserted between the upstream arm and downstream arms. All plasmid constructs were verified by DNA sequencing by the Keck DNA Facility (Yale University).

For transfection, S34 cells were dissociated into single cells by Accutase (STEMCELL Technologies, Cambridge, MA). Single S34 cells were plated on a Matrigel-coated 10 cm culture dish with mTeSR™1 medium (STEMCELL Technologies) plus 10.0 µM ROCK inhibitor, Y-27632 (STEMCELL Technologies)^48^. Then, the cultures were transfected with the Cas9-CLDN19-gRNA and linearized donor plasmids using Lipofectamine 3000 (Life Technologies), as directed by the manufacturer. Selection with puromycin was initiated when RFP-positive clones appeared. The initial concentration of puromycin was 0.5 μg ml^−1^, followed by additional rounds of selection until the concentration of puromycin reached 2.0 μg ml^−1^. The genotype of individual RFP+ clones was verified by DNA sequencing. The off-target analysis was performed on the top 10 high-risk regions predicted by the method of Labuhn et al.^49^.

### Differentiation of S34 human induced pluripotent cells into RPCs and RPE

Retinal neural differentiation was performed by modifying previous methods^50^. Briefly, at DD0, human induced pluripotent cells were dissociated into single cells by Accutase (STEMCELL Technologies). The single cells were suspended with STEMdiff™ Neural Induction Medium (STEMCELL Technologies) plus 10.0 µM ROCK inhibitor to obtain a final concentration of 1 × 10^5^ cells ml^−1^. The cell suspension (0.5 ml) was added to a well of the AggreWell™800 plate (STEMCELL Technologies). From DD1 to DD6, 50% of the medium was changed every day. On DD7, ~2000 embryoid bodies (EBs) were transferred to Matrigel-coated 10 cm culture dishes. Cells were assessed every day from DD8 to DD20 to monitor the formation of neural rosettes, and the medium replaced with STEMdiff™ Neural Induction Medium. At DD20, the neural rosettes were collected using STEMdiff™ Neural Rosette Selection Reagent (STEMCELL Technologies). Then, the selected neural rosettes were transferred on Ultra-low Attachment 6-well plates with retinal differentiation medium (DMEM/F12 3:1 mixture, 1× B27, 2.0 mM Glutamax, 1× non-essential amino acids, 1.0 mM sodium pyruvate, 1.0 mM taurine, 10.0 µM DAPT, and 10% FBS).

RPE was differentiated by a modification of our published method^13^. Briefly, optic vesicles were differentiated as described above. On DD20, the medium was switched to RPE differentiation medium (DMEM/F12, 1:1; 14% KSR; 1× non-essential amino acids; 2.0 mM Glutamax; 1.0 mM sodium pyruvate; 10.0 mM nicotinamide; and 100 ng ml^−1^ Activin A). From DD60 to DD80, pigmented RPE clumps were harvested and re-plated on Matrigel-coated plates. After two passages, confluent cultures were allowed to differentiate for 6 weeks prior to analysis^13^.

### Quantitative-real-time RT-PCR

The qRT^2^-PCR was performed as described previously^51^. For retinal organoids, total RNA was isolated from pools of 20–30 organoids. Briefly, 2.0 µg of total RNA was reverse-transcribed to cDNA (Quantitect Reverse Transcription Kit; Qiagen, Waltham, MA). Real-time RT-PCR was performed (iTaq Universal SYBR® Green SuperMix and Bio-Rad CFX 96 thermal cycler; Bio-Rad, Hercules, CA) following the manufacturer’s instructions. Custom PCR arrays for RPE and retinal development were prepared by Bio-Rad. Experiments were performed in triplicate with a minimum of two biological repeats. Glyceraldehyde 3-phosphate dehydrogenase (GAPDH) and beta-actin were used as controls to normalize the data. Relative expression of mRNA was calculated using the 2^−ΔΔCT^ method^52^.

### Microscopy

Live cell imaging was obtained using an Axiovert 40 CFL inverted microscope with fluorescence optics (Carl Zeiss, Thornwood, NY). For fixed tissues, (1) eye cups were prepared from enucleated eyes and fixed with 4% paraformaldehyde in phosphate-buffered saline (PBS) for 30 min at room temperature. The neurosensory retina was peeled from the eye cups, and post-fixed in 4% paraformaldehyde for another 30 min. The tissues were incubated in a series of graded sucrose solutions in PBS up to 30% sucrose and embedded in OCT (Sakura Finetek USA, Torrance, CA) for frozen sectioning. Sections (20 µm) were prepared with a Leica CM1850 cryostat. (2) Flat mounts of RPE were permeabilized with PBS containing 0.1% Triton X-100 prior to immunostaining. (3) Cultured cells were fixed in 2% paraformaldehyde in PBS for 10 min and permeabilized with Triton X-100.

For immunostaining, tissues or cultures were blocked with 5% normal donkey serum and incubated with primary antibodies (Supplementary Table 1) overnight at 4 °C followed by appropriate secondary antibodies. Secondary antibodies, raised in donkey and conjugated with Cy2, Cy3, or Cy5, were obtained from Jackson ImmunoResearch Laboratories (West Grove, PA). Nuclei were stained with 4′,6-diamidino-2-phenylindole (DAPI) and mounted by Fluoromount-G (SouthernBiotech, Birmingham, AL). TUNEL assays were performed on RPE flat mounts or retina sections by using Click-iT^®^ Plus TUNEL Assay kits (Thermo Scientific) following the instructions of the manufacturer. Confocal images were acquired using a LSM 410 spinning-disc confocal microscope. The images were processed, as described in Supplementary Methods, using Zen software (Carl Zeiss).

Relative thickness of the nuclear layers was calculated using Zen morphometry software at 20 locations in 4–5 mice. The length of bipolar axons and dendrites was measured by staining the cells with antibodies to protein kinase Cα (PKCα) and measuring the processes from the proximal edge of the bipolar cell nuclei to the end of the process, as described for measuring the thickness of nuclear layers. The density of TUNEL-positive nuclei was measured by number of positive nuclei found along a 500 µm length of outer nuclear layer in 4–5 mice.

### Plasmid construction and transfection

The coding sequence of wild type of CLDN19 was amplified from a cDNA library derived from human fetal RPE cells with paired primers that also added a FLAG-tag sequence (FLAG: atgGATTACAAAGACGATGACGATAAG). CLDN19 mutations were generated from this wild-type CLDN19 plasmid using PCR-based site mutagenesis and Gibson Assembly methods^53^. The hemagglutinin (HA) tag sequence (HA: atgTACCCATACGATGTTCCAGATTACGCT) was added to the primers for the 5’ end of the coding region of the CLDN19 mutations. All amplified sequences were then cloned downstream of a CAG promoter in AAV-CAG or pCAGGS vectors (Addgene)^46^. All constructs were verified by DNA sequencing (Keck DNA Facility). Plasmid transfections of ARPE-19 cells were performed using Lipofectamine 3000 (Life Technologies), as directed by the manufacturer.

### Production of recombinant adeno-associated viral vectors

High titer AAV2/5 particles were produced using AAV2 and AAV5 serotype plasmids at equal ratios and helper plasmid in HEK293 and purified on HiTrap Heparin HP (GE Healthcare). Briefly, when cultures were 80% confluent, HEK293 was transfected with the CLDN19 plasmid, pAAV2/5 serotype plasmid, and adeno virus helper plasmid using 1.0 mg ml^−1^ polyethylenimine. Culture medium was collected after 48 h and filtered through a 0.45 µm polyvinylidene difluoride filter (Millipore). The AAV virus was purified and concentrated using iodixanol gradient centrifugation and heparin affinity column (GE Healthcare Life Sciences, USA). Tittering of viral particles was done by qPCR^54^.

### Subretinal injection

Mouse pups were anesthetized by hypothermia on crushed ice. With the aid of a stereo dissection microscope (Leica/Wild M3Z, Buffalo Grove, IL), the eye was opened by dividing the fused junctional epithelium. Total 0.3–0.5 µl of rAAV particles (10^12^ ml^−1^) were slowly injected into the subretinal spaces using pre-pulled glass needle (P-97 Micropipette Puller, Sutter Instrument, Novato, CA). A mixture of viral particles was used that included AAV-GFP (to monitor the transfection efficiency) and AAV-CLDN19 (WT or mutant). Immunostaining was used to confirm claudin-19 expression. Upon recovery from the procedure, the pups were returned to their mother. The success rate for the viral injections was ~40–60%

### Fundus ophthalmoscopy and multifocal electroretinography

The mice were anesthetized by an intraperitoneal injection with ketamine/xylazine: 87.5 mg kg^−1^ ketamine (Hospira, Inc., Lake Forest, IL) and 12.5 mg kg^−1^ xylazine (Akorn, Inc., Lake Forest, IL). The dilation of mice pupil was performed with eyedrops of atropine sulfate (1%) and following of tropicamide (5 mg ml^−1^, USP). To prevent nystagmus, tetracaine (0.5%, USP) was used as a topical anesthetic. Mice were positioned on a warmed table and a fundus image was obtained using the confocal scanning laser ophthalmoscopy (cSLO) module of the RETImap system (Roland Consult, Brandenburg, Germany). Multifocal-electroretinography was obtained using the mfERG module of the RETImap system, as described previously^55^. Briefly, a DTL thread electrode was placed along the corneal limbus of mouse eye and covered with a contact lens. Viscous methocel, 2% gel (OmniVision GmbH, Puchheim, Germany) was applied underneath the contact lens. Subcutaneous silver needle electrodes served as reference and ground electrodes. The responses were amplified ×100,000 and a 60 Hz band-pass filtered was applied. The signals were digitized and acquired with 1024 Hz sampling frequency.

For the retinoid-rescue experiment, mfERG recordings were obtained for wild-type mice and mice transduced with CLDN19 mutations. The mice (4–5 mice for each condition) were allowed for recover for 1 week in normal light/dark cycle environment. Mice were then dark-adapted overnight in preparation for treatment with retinoids. 9-*cis*-retinal or all-*trans*-retinal (Sigma-Aldrich) was diluted in 200 μl carrier solution (10% ethanol, 10% bovine serum albumin in 0.9% NaCl). Each mouse received an intraperitoneal injection of 0.25 mg under dim red light. The retinal-treated mice were kept in the dark for 1 day followed by photopic, multifocal electroretinogray recordings the next day.

### Statistics

The significance of statistical data was estimated using the unpaired, two-tailed version of Student's *t*-test or one-way analysis of variance.

### Reporting Summary

Further information on experimental design is available in the Nature Research Reporting Summary linked to this article.

## Supplementary information

Reporting Summary

Supplemental Information

Supplementary Data 1

Description of Supplementary Data 1

## Data Availability

The authors affirm that the data that support the findings of this study are found within the paper or in supplementary information and supplementary data 1. Clones generated by this study are available from the authors with the consent of WiCell and Dr. Donald Zack, Johns Hopkins University.
