# Supplementary material for: Disease-associated mutations of claudin-19 disrupt retinal neurogenesis and visual function
Source: Commun Biol. 2019 Mar 25;2:113. doi: 10.1038/s42003-019-0355-0 (PMC6433901; doi:10.1038/s42003-019-0355-0)
Supplement: Supplementary file 1 — Reporting Summary [file 42003_2019_355_MOESM1_ESM.pdf]

nature research

Corresponding author(s): Lawrence J Rizzolo

Reporting Summary

Nature Research wishes to improve the reproducibility of the work that we publish. This form provides structure for consistency and transparency in reporting. For further information on Nature Research policies, see Authors & Referees and the Editorial Policy Checklist.

Statistical parameters

When statistical analyses are reported, confirm that the following items are present in the relevant location (e.g. figure legend, table legend, main text, or Methods section).

n/a	Confirmed	
☐	☒	The exact sample size (*n*) for each experimental group/condition, given as a discrete number and unit of measurement
☐	☒	An indication of whether measurements were taken from distinct samples or whether the same sample was measured repeatedly
☐	☒	The statistical test(s) used AND whether they are one- or two-sided<br>*Only common tests should be described solely by name; describe more complex techniques in the Methods section.*
☒	☐	A description of all covariates tested
☒	☐	A description of any assumptions or corrections, such as tests of normality and adjustment for multiple comparisons
☐	☒	A full description of the statistics including central tendency (e.g. means) or other basic estimates (e.g. regression coefficient) AND variation (e.g. standard deviation) or associated estimates of uncertainty (e.g. confidence intervals)
☐	☒	For null hypothesis testing, the test statistic (e.g. *F*, *t*, *r*) with confidence intervals, effect sizes, degrees of freedom and *P* value noted<br>*Give P values as exact values whenever suitable.*
☒	☐	For Bayesian analysis, information on the choice of priors and Markov chain Monte Carlo settings
☒	☐	For hierarchical and complex designs, identification of the appropriate level for tests and full reporting of outcomes
☒	☐	Estimates of effect sizes (e.g. Cohen's *d*, Pearson's *r*), indicating how they were calculated
☐	☒	Clearly defined error bars<br>*State explicitly what error bars represent (e.g. SD, SE, CI)*

Our web collection on statistics for biologists may be useful.

Software and code

Policy information about availability of computer code

Data collection	Multifocal Electroretinogram: RetiMap, Roland Consult, Brandenburg, Germany; Confocal microscopy: Zen data acquisition and analysis, Zeiss, Thornwood, NY
Data analysis	Same as data collection; Prism, statistics and graphing program

For manuscripts utilizing custom algorithms or software that are central to the research but not yet described in published literature, software must be made available to editors/reviewers upon request. We strongly encourage code deposition in a community repository (e.g. GitHub). See the Nature Research guidelines for submitting code & software for further information.

Data

Policy information about availability of data

All manuscripts must include a data availability statement. This statement should provide the following information, where applicable:

- Accession codes, unique identifiers, or web links for publicly available datasets
- A list of figures that have associated raw data
- A description of any restrictions on data availability

The authors affirm that the data that support the findings of this study are found within the paper or in Supplementary Information and Supplementary Data 1.

Field-specific reporting

Please select the best fit for your research. If you are not sure, read the appropriate sections before making your selection.

☒ Life sciences ☐ Behavioural & social sciences ☐ Ecological, evolutionary & environmental sciences

For a reference copy of the document with all sections, see nature.com/authors/policies/ReportingSummary-flat.pdf

Life sciences study design

All studies must disclose on these points even when the disclosure is negative.

Sample size	iPSC: For each experimental group, 2-3 clones were analyzed in depth. Cell cultures (hfRPE and ARPE-19): experiments were repeated three times. Mouse experiments: repeated 3 times with three mice
Data exclusions	No data was excluded.
Replication	Both biological (independent experiments) and technical (same sample quantified multiple times) repeats were performed for each study. experiment.
Randomization	For mouse studies, entire cages (5 animals per cage per University requirements) were assigned to an experimental group). The animals in the cage came from the same liter. Generally, the success rate for viral infections was 60%
Blinding	Only discrete variables were measured and the experimental design minimized bias in the choice of samples that were measured

Reporting for specific materials, systems and methods

Materials & experimental systems

n/a	Involved in the study
☐	☒ Unique biological materials
☐	☒ Antibodies
☐	☒ Eukaryotic cell lines
☒	☐ Palaeontology
☒	☐ Animals and other organisms
☒	☐ Human research participants

Methods

n/a	Involved in the study
☒	☐ ChIP-seq
☒	☐ Flow cytometry
☒	☐ MRI-based neuroimaging

Unique biological materials

Policy information about availability of materials

Obtaining unique materials	All unique materials are available from the authors.

Antibodies

Antibodies used	All antibodies were from commercial sources and described in Supplemental Table 1.
Validation	Validation is available on the manufacturer's website for use in mouse and human tissue.

Eukaryotic cell lines

Policy information about cell lines

Cell line source(s)	ARPE-19: ATCC; S34 derivative of IMR90-4 (WiCell. Madison, WI; gift of Karl Wahlin and Donald Zack (Johns Hopkins Univ)
Authentication	STR testing by ATCC
Mycoplasma contamination	Cultures tested negative using the Mycosensor QPCR Kit (Agilent, Santa Clara, CA)

Commonly misidentified lines<br>(See ICLAC register)	None
