# Supplementary material for: Disease-associated mutations of claudin-19 disrupt retinal neurogenesis and visual function
Source: Commun Biol. 2019 Mar 25;2:113. doi: 10.1038/s42003-019-0355-0 (PMC6433901; doi:10.1038/s42003-019-0355-0)
Supplement: Supplementary file 2 — Supplemental Information [file 42003_2019_355_MOESM2_ESM.pdf]

Supplementary Figures

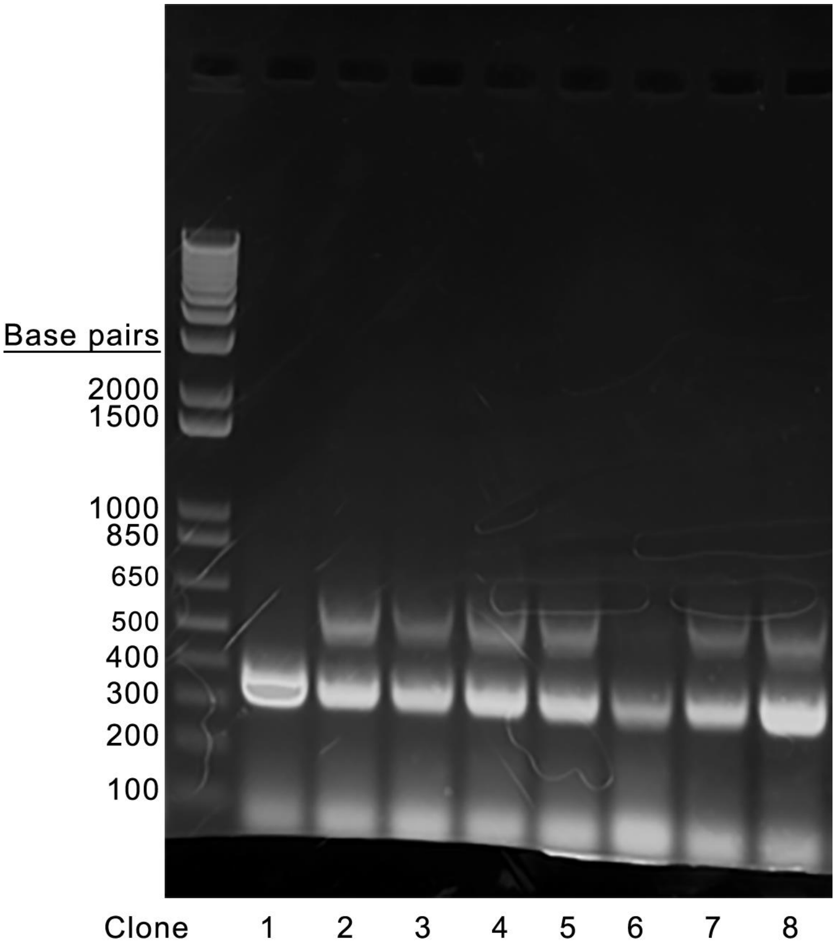

Supplementary Figure 1. Uncropped gel for Figure 1B. Standards at the left are 1 Kb plus DNA ladder, Invitrogen.

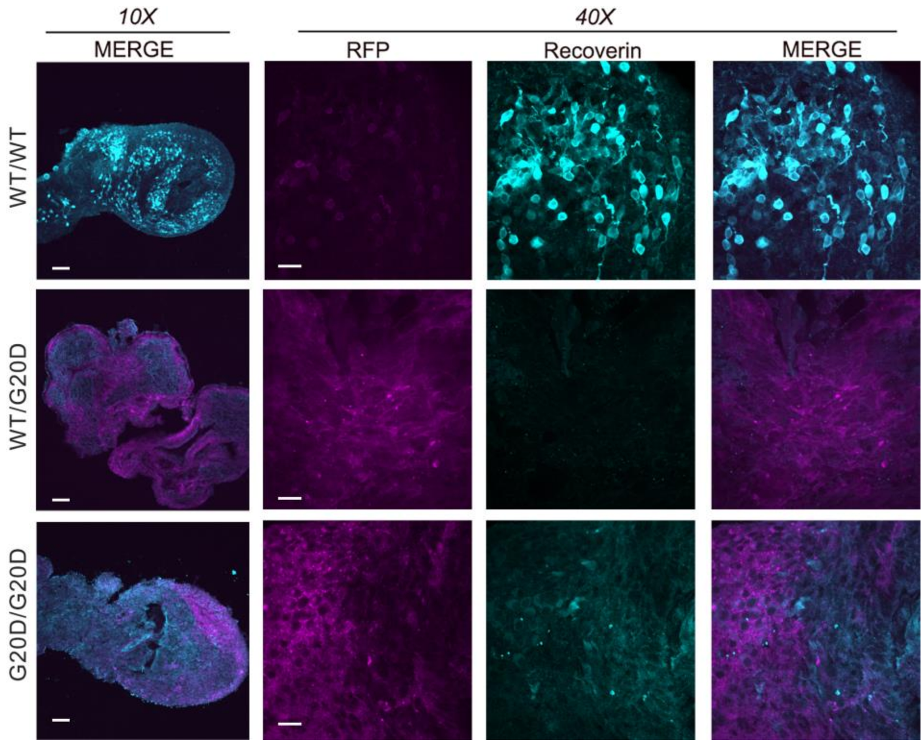

Supplementary Figure 2. Retinal cups derived from $CLDN19^{WT/G20D}$ or $CLDN19^{G20D/G20D}$ hiPSC failed to express the photoreceptor marker, recoverin. On DD80, retinal cups were immunostained to reveal the expression of recoverin. RFP-puromycin revealed transduced hiPSC. Low power views in the left column were acquired with a 10x objective to demonstrate retinal cups for $CLDN19^{WT/WT}$ and $CLDN19^{WT/G20D}$ cultures, but non-descript structures for $CLDN19^{G20D/G20D}$. High power views were acquired with a 40x-oil objective to demonstrate the presence of recoverin in $CLDN19^{WT/WT}$, but not $CLDN19^{WT/G20D}$ or $CLDN19^{G20D/G20D}$ cultures. Scale Bars: 10× panels, 40 µm; 40× panels, 20µm

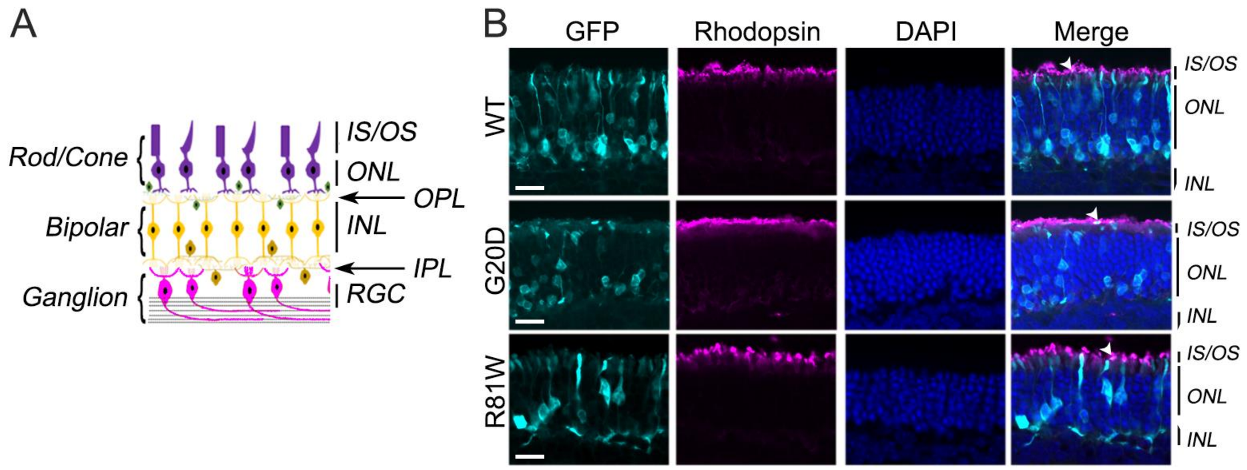

Supplementary Figure 3. Expression of CLDN19 or its mutants did not affect the formation of photoreceptor inner and outer segments. Mice were injected on PN0 and eyes harvested on PN30. Antibodies to Rhodopsin reveal the distribution of un-transduced and transduced photoreceptors. A) Schema of the retina. B) GFP (cyan) marks transduced cells. The white signal in the merged images (arrowheads) indicates the co-localization of rhodopsin and GFP. IS, photoreceptor inner segment; OS, photoreceptor outer segment; ONL, outer nuclear layer; OPL, outer plexiform layer; INL, inner nuclear layer; IPL, inner plexiform layer; RGC, retinal ganglion cell layer; Rhodopsin, magenta; DAPI, Blue; Scale bar, 20 μm

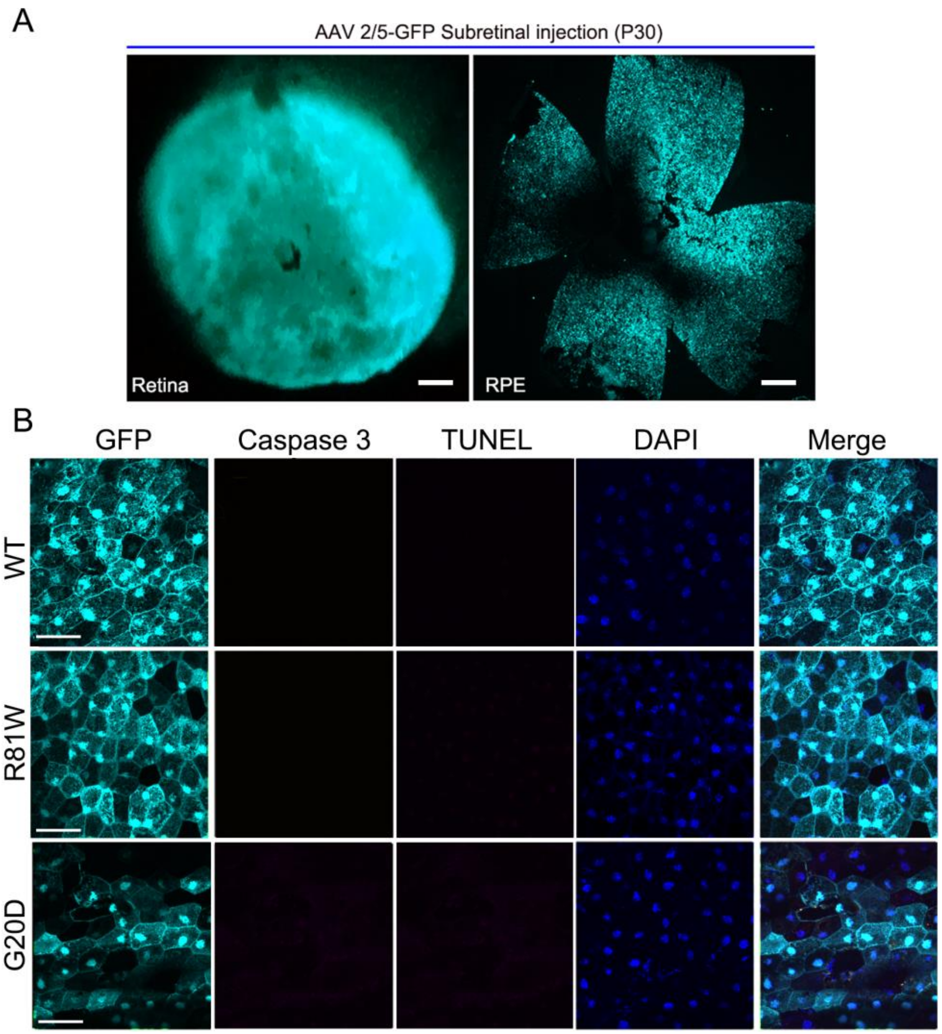

Supplementary Figure 4. Mutated Claudin-19 did not induce cell apoptosis in mouse RPE. GFP indicates cells transduced by the AAV viral vector. Images were obtained on PN30. A) Flat-mount images of the retina and RPE (after the neurosensory retina was removed) indicate the extent of the retina that was infected by the viral vector. Scale Bar 500 µm B) In higher power images of the RPE, a fluorescence signal was not observed due to the TUNEL assay or for immunofluorescence labeling of the cleaved form of caspase 3. Scale Bar, 20 µm

Supplementary Table 1: Details of Primary antibodies

Antigen	Host[1]	Catalogue	Dilution	Supplier
BRN3	MM	sc-390780	1:50	Santa Cruz Biotechnology
Caspase-3	RP	9662	1:1000	Cell Signaling Technology
Claudin-19	MM	MAB6970	10 μg/ml	R&D Systems
FLAG-tag	MM	9A3	1:1600	Cell Signaling Technology
HA-tag	RM	C29F4	1:1600	Cell Signaling Technology
N-Cadherin	MM	C3865	2 μg/ml	Sigma Aldrich
OCT4	RP	2890S	1:200	Cell Signaling Technology
PKCα	RM	A302-446A	1:100	Abgent
Recoverin	RP	AB5585	1:500	EMD Millipore
Rhodopsin	MM	R5403	1:400	Cell Signaling Technology
RPE65	MM	MAB5428	1:400	EMD Millipore
SSEA4	MM	4744S	1:500	Cell Signaling Technology
ZO-1	RM	D7D12	1:500	Life Technologies

[1]RP, Rabbit Polyclonal; RM, Rabbit Monoclonal; MM, Mouse Monoclonal; MP, Mouse Polyclonal; SP, Sheep Polyclonal; GP, Goat Polyclonal

Supplementary Methods

Imaging Studies
For all confocal microscopic images, black and white images for each channel were acquired using Zen Software (Zeiss, Thornwood, NY) and false-colored: cyan for GFP/Cy2, magenta for RFP/Cy3, orange for Cy5, and blue for DAPI. White on the images indicates the overlap of the cyan and magenta signals. The same linear adjustments (gamma=1.00) were applied to all images in the same panel using the settings indicated in for each figure below. "White" settings were determined for the brightest sample and applied to all panels in the figure including the control. The settings used for each Figure Panel are included below. Post-processing in Photoshop was confined to expanding levels for the entire image such that all data of the brightest image was included in the range 0-255, and that setting applied for all images in the panel.

<u>LSM 410 spinning-disc confocal microscope specifications:</u>
Lasers: 405nm, 488nm, 532/561nm, 639nm

Filter set	Item number	Excitation	Beam splitter	Emission
Filter Set 38 HE (FITC/GFP)	489038-9901-000	BP 470/40 (HE)	FT 495 (HE)	BP 525/50 (HE)
Filter Set 43 HE (dsRED/Rhodamine)	489043-9901-000	BP 550/25 (HE)	FT 570 (HE)	BP 605/70 (HE)
Filter Set 49 (DAPI)	489049-9901-000	G 365	FT 395	BP 445/50
Filter Set 50 (Cy 5)	489050-9901-000	BP 640/30	FT660	BP 690/50

Axiovert 40 CFL inverted microscope filter sets (Omega Optical)

	XF404	XF406
Excitation	450-486 nm	532-576 nm
Emission	510-558 nm	598-645 nm

Figure 1:
Panel D: Zen window settings

Channel	Black	Gamma	White
DAPI	30	1.0	1115
Cy2/GFP	50	1.0	1436
Cy3/RFP	30	1.0	994
Cy5	30	1.0	1280

Figure 2

Panel B. Image acquired with an Iphone mounted on an Axiovert 40 CFL inverted microscope with no post-processing.

Panel C: Zen window settings

Channel	Black	Gamma	White
DAPI	0	1.0	1266
Cy2/GFP	0	1.0	393
Cy3/RFP	0	1.0	1004

Figure 3

Panel A: Zen window settings

Channel	Black	Gamma	White
DAPI	100	1.0	4096
Cy3/RFP	0	1.0	500
Cy5	0	1.0	1485

Figure 4

Panel B: Image acquired with an Iphone and no post-processing.

Figure 5

Panel B: Zen window settings

Channel	Black	Gamma	White
Cy2/GFP	47	1.0	208
Cy3/RFP	0	1.0	431
DIC	0	1.0	1708

Figure 6

Panel B: Zen window settings

Channel	Black	Gamma	White
DAPI	20	1.0	1565
Cy2/GFP	20	1.0	382
Cy3/RFP	20	1.0	482

Panel C: Zen window settings

Channel	Black	Gamma	White
Cy2/GFP	20	1.0	621
Cy3/RFP	20	1.0	184

Panel D: RetiMap confocal scanning laser ophthalmoscope (cSLO) image with no post-processing.

Figure 7

Panel B: Zen window settings

Channel	Black	Gamma	White
DAPI	0	1.0	2600
Cy2/GFP	0	1.0	2908
Cy5	0	1.0	2050

Panel E: Zen window settings

Channel	Black	Gamma	White
DAPI	0	1.0	2600
Cy2/GFP	0	1.0	761
Cy3/RFP	0	1.0	1000
Cy5	0	1.0	1325

Figure 8

Panel A: Zen window settings:

Channel	Black	Gamma	White
Cy2/GFP	0	1.0	2375
Cy3/RFP	0	1.0	408

Panel C: Zen window settings Channels matched to highlight double labeling in the merged image.

Wild type

Channel	Black	Gamma	White
DAPI	0	1.0	1200
Cy2/GFP	0	1.0	770
Cy3/RFP	0	1.0	584

R81W

Channel	Black	Gamma	White
DAPI	0	1.0	1200
Cy2/GFP	0	1.0	873
Cy3/RFP	0	1.0	889

G20D

Channel	Black	Gamma	White
DAPI	0	1.0	1200
Cy2/GFP	0	1.0	3530
Cy3/RFP	0	1.0	1084

Supplementary Figure 1

Zen window settings: Note that Cy5 is false colored green in this figure

10X column

Channel	Black	Gamma	White
Cy3/RFP	0	1.0	547
Cy5	0	1.0	1500

40X columns

Channel	Black	Gamma	White
Cy3/RFP	0	1.0	760
Cy5	0	1.0	3400

Supplementary Figure 2

Zen window settings

Channel	Black	Gamma	White
DAPI	0	1.0	3280
Cy2/GFP	0	1.0	742
Cy3/RFP	0	1.0	2423

Supplementary Figure 3

Panel A: Image acquired with an Iphone and no post-processing.

Panel B: Zen window settings

Channel	Black	Gamma	White
DAPI	0	1.0	901
Cy2/GFP	0	1.0	2400
Cy3/RFP	0	1.0	1800
Cy5	0	1.0	1800
