# Supplementary material for: Disease-associated mutations of claudin-19 disrupt retinal neurogenesis and visual function
Source: Commun Biol. 2019 Mar 25;2:113. doi: 10.1038/s42003-019-0355-0 (PMC6433901; doi:10.1038/s42003-019-0355-0)
Supplement: Supplementary file 4 — Description of Supplementary Data 1 [file 42003_2019_355_MOESM4_ESM.docx]

Description of additional supplementary items:

File name: Supplementary Data 1

File type: Excel

Description: Source data underlying Figures 3-9.
